# Prevalence of Being Obese, Overweight, and Underweight Among Jordanian Children and Adolescents Based on International Growth Standards

**DOI:** 10.3390/healthcare13020146

**Published:** 2025-01-14

**Authors:** Walid Al-Qerem, Ruba Zumot, Anan Jarab, Judith Eberhardt, Fawaz Alasmari, Alaa Hammad

**Affiliations:** 1 Department of Pharmacy, Faculty of Pharmacy, Al-Zaytoonah University of Jordan, Amman 11733, Jordan; waleed.qirim@zuj.edu.jo (W.A.-Q.); 202127091@std-zuj.edu.jo (R.Z.); alaa.hammad@zuj.edu.jo (A.H.); 2 College of Pharmacy, Al Ain University, Abu Dhabi 112612, United Arab Emirates; anan.jarab@aau.ac.ae; 3 AAU Health and Biomedical Research Center, Al Ain University, Abu Dhabi 112612, United Arab Emirates; 4 Department of Clinical Pharmacy, Faculty of Pharmacy, Jordan University of Science and Technology, Irbid 22110, Jordan; 5 Department of Psychology, School of Social Sciences, Humanities and Law, Teesside University, Borough Road, Middlesbrough TS1 3BX, UK; 6 Department of Pharmacology and Toxicology, College of Pharmacy, King Saud University, Riyadh 12372, Saudi Arabia; ffalasmari@ksu.edu.sa

**Keywords:** Jordanian children, obesity, prevalence, underweight

## Abstract

Objectives: The rise of obesity and other nutrition-related conditions among children and adolescents is a global challenge, particularly in the Middle East. This study aimed to determine the prevalence of being underweight, overweight, and obese among Jordanian children and adolescents using the body mass index (BMI) percentiles of the World Health Organization (WHO) and Centers for Disease Control and Prevention (CDC) standards. Methods: This retrospective cross-sectional/longitudinal study analyzed 58,474 (42.6% males; 57.4% females) height, weight, and BMI-for-age records from 31508 healthy Jordanian children and adolescents aged 2–19 years. The data were retrieved from the Ministry of Health’s nationwide electronic database (2017–2023) and assessed using the CDC and WHO growth standards. Logistic regression was performed to assess the variables associated with overweight/obese status. Results: The prevalence of being underweight, overweight, and obese varied by the reference used, as more cases of being obese and underweight were reported when applying the CDC standards. The regression models showed the males had significantly lower odds of being overweight and obese than the females. Increased age was associated with higher odds of being overweight and obese, with annual increases observed across all age groups. Conclusions: Using the WHO and CDC standards, the prevalence of being underweight was higher in the males aged 6 years and older, while being overweight and obese was more prevalent in the females. The observed annual increase in the prevalence of being overweight and obese underscores the need for targeted strategies. Growth references tailored to regional profiles may improve national nutrition policies for Jordanian children and adolescents.

## 1. Introduction

Children’s weight and height are key growth indicators allowing healthcare providers to identify potential underlying illnesses in seemingly healthy individuals [1]. In childcare, it is essential to evaluate the growth of children and adolescents using objective and noninvasive anthropometric measures such as weight, height, and body mass index (BMI). These measures provide fundamental insights into growth status, accurately indicating their nutritional status, health, and well-being. Additionally, they help screen for underlying growth defects, diseases, and various social or nutritional problems [1]. Multiple forms of childhood malnutrition, such as being underweight, overweight, or obese, contribute to an increase in global health problems, which impact both developed and developing nations equally [2]. These conditions have been linked to Type 2 diabetes, high blood pressure, heart disease, anemia, weakened immunity, and psychological disorders. They can also hinder children’s [3] and adolescents’ academic performance [4]. Growth references are widely used and are valuable in assessing children’s health and well-being [5]. They help mitigate disputes regarding abnormal growth and development, foster healthy growth and development, and aid in the detection of underlying diseases. Standardized growth charts compare a child’s measurements to peers of the same age and sex, helping assess healthy growth and determine normalcy. Among the most widely used and useful anthropometric measurements is the child’s weight, which responds well to acute effects. Following this are head circumference and height, with height being a significant parameter that can help diagnose and potentially prevent stunting in early life [6]. Early detection of growth abnormalities allows for treatment to minimize their impact and optimize a child’s final adult stature [1]. Using reference percentiles for weight, height, and BMI aids in assessing health problems in children. For instance, calculating BMI and comparing it to the CDC’s BMI percentiles helps classify children and adolescents as underweight, of normal weight, overweight, or obese [7].

BMI is a widely used screening tool for weight status, valued for its simplicity in calculation and application. The weight status of children and adolescents is assessed using international growth percentiles developed by the WHO and CDC, which account for variations in body composition by sex and age. Despite its widespread use, BMI has certain limitations. For instance, it does not account for the rapid changes that occur during growth spurts, which can result in an overestimation of being overweight or obese in young children. Furthermore, BMI does not differentiate between muscle mass and body fat, leading to inaccurate conclusions for athletic children or those with low muscle mass [8].

Insufficient studies have been conducted to examine the trends and prevalence of being underweight, overweight, and obese, along with their associated factors, in the Jordanian population. However, an increase in the prevalence of being overweight and obese has been observed among Jordanian children and adolescents. A 2017 study reported a rising rate of obesity across the Jordanian population [9]. Additionally, resources such as the Global Obesity Observatory, an online dataset, provide information about the prevalence of being overweight and obese, as well as trends over time by country or region [10]. The complexity of weight-related disorders highlights a double burden of malnutrition. A comprehensive approach that examines how these disorders affect different demographics, such as age and gender, is strongly recommended in order to develop tailored therapeutic strategies aimed at prevention and improving overall health in Jordan.

Therefore, utilizing international standards for comparison, the present study aimed to determine the prevalence rates of being underweight, overweight, and obese, as well as trends in being overweight and obese among Jordanian children and adolescents over the past six years (2017–2023). This analysis aimed to provide a foundation for further research and policy development focused on improving nutritional health within this demographic.

## 2. Materials and Methods

### 2.1. Study Design and Participants

In this retrospective longitudinal and cross-sectional study, participants’ data were retrieved from the Hakeem database, a nationwide electronic health information system for the Jordanian population registered with the Ministry of Health and the Royal Medical Services. The dataset contained records of children and adolescents from the years 2017, 2019, 2021, and 2023. It contained participants’ identification number (ID), gender, weight, height, medical history, date of birth, and date and location of measurement; the age of each participant at the time of measurement and their BMI were calculated, respectively. The measurement units used were standardized: height in cm, weight in kg, and BMI in kg/m^2^. The selected data, which complied with the CDC selection criteria, included Jordanian children and adolescents aged 2–19 years.

For age- and gender-related analyses, the data were subgrouped by gender and categorized into sequential age intervals (2–5 years, 6–11 years, and 12–19 years) [11,12]. Height for age, weight for age, and body mass index for age were analyzed comparatively for the Jordanian children and adolescents against the CDC and WHO growth references.

### 2.2. Sample Size Calculation

The retrieved data were collected from different geographic locations in Jordan to ensure the generalizability of the results. The proportion of data retrieved by location (city/governate) was proportional to the population distribution of the study age group. Based on previously published WHO figures [13], the expected prevalence of obesity was determined to be 12.9%. The required sample size, calculated to achieve a margin of error or absolute precision of ±1% in estimating the prevalence with 95% confidence, was 4317. With this sample size, the anticipated 95% CI was (11.9–13.9%). The sample size was calculated using the Scalex SP calculator [14]. The study retrieved a much larger dataset to enhance the validity and reliability of the results, even after data cleaning and the exclusion of ineligible records.

### 2.3. Statistical Analysis

Continuous variables were presented as medians (25th–75th percentiles) and categorical variables were presented as frequencies (%). The Related-Samples Marginal Homogeneity Test was used across all age groups and both sexes to evaluate the distribution of weight status based on the CDC and WHO standards. Statistical analyses were conducted employing IBM SPSS Statistics^®^, Version 26 [15]. Binary regression models were applied to assess the variables associated with obesity prevalence based on the WHO and CDC equations. Two sets of regression models were used: the first included the year of the anthropometric record, sex, and age group, while the second, conducted separately for each age group, included age, year of anthropometric record, and sex.

### 2.4. Growth References

Nutritional status indicators were determined using both the CDC and WHO growth references. Following the CDC’s classification system, which applies to individuals aged 2 to 20 years, the 5th and 95th percentiles serve as cutoff values for identifying abnormal growth and malnutrition. This system uses age- and sex-specific percentiles, categorizing BMI-for-age below the 5th percentile as underweight. BMI values between the 5th and just below the 85th percentile are considered normal or healthy weight, those between the 85th and 95th percentile are classified as overweight, and values at or above the 95th percentile are classified as obese [16] (See Table 1).

Applying the WHO’s global classification, which uses the 3rd and 97th percentiles as the outermost cutoff points to identify abnormal growth, the age groups were divided into two sets.

Growth indicators for children younger than 5 years were defined in the following manner: a weight-for-age value below the 3rd percentile was considered underweight; a BMI-for-age value above the 97th percentile but below the 99.9th percentile was classified as overweight; and a BMI-for-age value above the 99.9th percentile was classified as obese [17,18,19,20].

For children aged 5 to 19 years, a weight-for-age value below the 3rd percentile was considered underweight; a BMI-for-age value above the 85th percentile but below the 97th percentile was classified as overweight; and a BMI-for-age value above the 97th percentile was classified as obese [21,22,23]. (See Table 2).

## 3. Results

**Figure 1:**
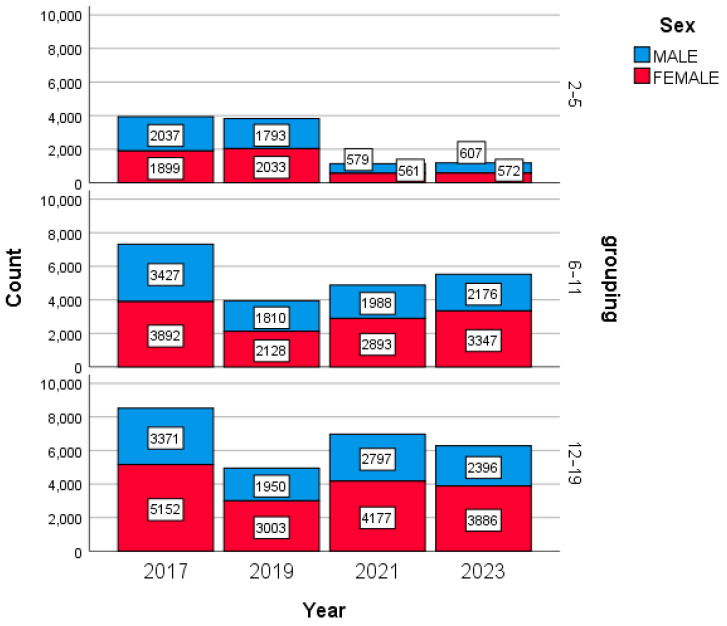
Anthropometric measure distribution between sexes, age groups, and different years.

A total of 58,474 anthropometric measures, 24,931 (42.6%) males and 33,543 (57.4%) females, were collected from 31,508 eligible participants (58.6% females); Figure 1 illustrates the distribution of the anthropometric measures by sex, age group, and year.

After grouping the participants by sex and age, the median values for age, height, weight, and BMI were calculated for each subgroup. The males generally had higher median heights and weights than the females in the 6–11 and 12–19 age groups, while the females exhibited slightly higher median BMIs in the older age groups. These trends highlight differences in the growth patterns between the sexes across various age groups (see Table 3).

Abnormal growth prevalence among the Jordanian children and adolescents from 2017 to 2023 was assessed using CDC and WHO growth references. The prevalence of being underweight, overweight, and obese varied between the two references across all the age groups and years. Generally, the CDC criteria yielded higher estimates for being underweight and obese, while the WHO reference reported higher overweight prevalence in several age groups. Notably, the males in early childhood (2–5 years) exhibited higher underweight prevalence than the females, whereas the females in adolescence (12–19 years) consistently had higher rates of being overweight and obese (see Table 4).

The Related-Samples Marginal Homogeneity Test revealed significant differences in weight status classifications between the CDC and WHO standards across all the age groups and sexes (*p* < 0.001). Multiple binary regression models identified the variables significantly associated with being overweight and obese in the Jordanian children and adolescents.

Using the WHO standards, the prevalence of being overweight and obese increased over the years, with the highest rates observed in the most recent readings (OR = 1.037). The females had higher odds of being in the significantly overweight and obese group compared to the males (OR = 0.662), and the older age groups exhibited a greater prevalence (OR = 1.352). Similar trends were observed when using the CDC standards, with slightly different odds ratios (see Table 5 and Table 6).

For the 2–5 age group, the males had higher odds of being overweight and obese compared to the females (OR = 1.133), but this was reversed in the older age groups. In both the 6–11 and 12–19 age groups, the females consistently exhibited higher prevalence rates, and being overweight and obese increased with age and over the years (ORs ranging from 1.035 to 1.115).

The findings highlight consistent upward trends in the prevalence of being overweight and obese across all the age groups and years, regardless of the classification system used, with notable differences between the sexes and age groups.

## 4. Discussion

The present study aimed to estimate the prevalence of being underweight, overweight, and obese among Jordanian children and adolescents aged 2–19 years, examine the trends in being overweight and obese from 2017 to 2023 and within each subgroup, and detect differences in prevalence based on the CDC and WHO references. This is the first study to assess the weight status of a large, diverse sample of participants from various governorates across the Kingdom of Jordan. It is also the first national study to estimate the prevalence of being underweight, overweight, and obese, as well as trends in being overweight and obese, utilizing both the CDC and WHO standards.

The findings suggest that the choice of BMI reference plays a major role in decision-making regarding appropriate medical interventions and treatments. It also affects estimates of the healthcare resources required to address being underweight, overweight, and obese. The main findings indicate that using the CDC criteria to assess the weight status of Jordanian children and adolescents results in higher estimates of being underweight and obese, but lower estimates of being overweight compared to the WHO criteria. This significant discrepancy highlights the impact of the chosen reference standard on prevalence assessments. The practical implications of this finding are substantial, as variations in prevalence estimates could influence public health priorities, resource allocation, and intervention strategies. A unified approach that integrates both global and regional growth standards may help minimize these discrepancies and provide a clearer picture of childhood malnutrition. Such variations emphasize the need for consensus on standardized tools that ensure consistent reporting and allow for meaningful cross-country comparisons. Moreover, these discrepancies could impact clinical decision-making, potentially leading to under- or over-diagnosis of malnutrition, particularly in resource-limited settings.

The prevalence of being underweight was more pronounced in the males, while the females exhibited higher rates of being overweight and obese according to both references. This finding aligns with a study conducted in seven African countries, which estimated the prevalence of being underweight and obese among schoolchildren aged 11–17 years using the WHO references. The study reported that the males had a higher prevalence of being underweight than females across all countries, while the females were more likely to be overweight at all ages in nearly all seven countries [24]. Another study comparing the prevalence of being underweight and overweight/obese among Nigerian children aged 6–16 years found that a higher percentage of the males were underweight compared to the females, while the females were more likely to be overweight or obese than the males [25]. These results align with findings from Saudi Arabia and Kuwait, where the prevalence of obesity among adolescents has been reported at 15–20% and up to 40–49%, respectively. Both studies highlighted that the females consistently had higher obesity rates than the males [26,27]. The observed trends in Jordan parallel these findings, suggesting shared regional risk factors such as dietary habits, sedentary lifestyles, and sociocultural influences. The higher prevalence of being underweight in boys may stem from biological differences, such as higher metabolic rates and greater physical activity levels, which increase energy expenditure. Additionally, cultural norms and feeding practices might result in boys receiving less attention to their nutritional needs in some contexts [28]. Additionally, societal norms and expectations around body image may disproportionately affect females, contributing to higher obesity rates. Addressing these factors could be key to reducing the gender disparities in obesity prevalence.

The observed variations in BMI values with increasing age and between genders highlight the normal changes in body composition that occur during puberty. Typically, the growth spike reflecting the pubertal spurt begins earlier in females than in males [29,30]. Comparable trends have also been observed in the United States, where the obesity rate among children and adolescents aged 2–19 years was 19.2% in 2017–2018. This rate is similar to the prevalence observed in older Jordanian adolescents, further emphasizing the global nature of this challenge [11]. The consistency of these trends across different populations highlights the role of shared global factors, such as urbanization and dietary transitions, in driving the rise of childhood obesity. A longitudinal study from Spain further underlines the influence of sociodemographic factors on weight status, noting that disparities in access to resources and healthcare services play a critical role in shaping childhood obesity trends [31]. The consistency of these trends across different populations underscores the role of shared global factors, such as urbanization and dietary transitions, in driving the rise of childhood obesity. This highlights the need for both local and international collaboration in addressing this public health issue. This finding should be given consideration, as using different references can lead to disparities in prevalence rates and potentially cause diagnostic bias. Similar studies examining the rates of agreement between the two references have yielded varied results. A systematic review examined 27 articles comparing the international BMI ratings, including those using the CDC and the WHO references. For instance, a study in Iran highlighted significant differences between the CDC and WHO references in estimating being overweight and obese among participants aged 11–15 years. Using the CDC reference, 8.5% of the males and 10% of the females were categorized as being overweight, compared to 10% of the males and 7.9% of the females according to the WHO reference. A more pronounced difference was observed in estimating obesity. Based on the CDC reference, 5.7% of the males and 10% of the females were categorized as obese, while the WHO reference categorized 8.5% of the males and 7.9% of the females as obese [32]. The sex differences were confirmed by the multiple binary regression models, which showed that the males had significantly lower odds of being in the overweight or obese group compared to the females. The regression models also showed that being overweight and obese were positively associated with increasing age, with each successive year exhibiting higher prevalence rates than the previous one. This observed year-on-year increase suggests a need for proactive measures, such as early intervention programs and public awareness campaigns, to mitigate the rising burden of obesity in Jordan. Future research should explore specific factors that could be targeted in these high-risk groups.

Since this study included schoolchildren from various districts in Jordan, its findings can be generalized to all Jordanian children and adolescents. Additionally, the large sample size enabled detailed subgroup analysis, providing a deeper understanding of gender-related differences in the prevalence of weight status among children and adolescents across different age groups.

Still, several limitations of this study need to be recognized. First, the lack of socioeconomic data hindered analysis of their impact on growth and development patterns. Moreover, the study’s design also imposed restrictions; its observational aspect precluded examining causal relationships between the variables, and the longitudinal section did not encompass the entire sample nor maintain consistent age intervals for measurements.

The study period (2017–2023) included the COVID-19 pandemic, which has been associated with increased childhood obesity due to factors like reduced physical activity and altered eating habits during lockdowns [33]. This context may have influenced our findings, potentially contributing to the observed rise in the rates of being overweight and obese among Jordanian children and adolescents. Future research should consider the pandemic’s impact when interpreting trends in weight status.

Future research should aim to incorporate comprehensive socioeconomic data to better understand contextual factors influencing growth and development. Longitudinal studies with consistent sampling and age intervals are also recommended to establish causal links and track changes over time. Furthermore, investigating the role of dietary habits, physical activity, and genetic predispositions may provide more profound insights into the underlying causes of weight-related issues. Tailored interventions that consider the cultural, economic, and social dynamics of Jordan could also play a pivotal role in addressing childhood malnutrition effectively.

## 5. Conclusions

This study found that, based on both CDC and WHO references, underweight prevalence was higher in Jordanian male children, while being overweight and obese were more prevalent in female children. However, the two references yielded different prevalence estimates across age groups, sexes, and years.

An annual increase in obesity prevalence is evident, highlighting the need for a comprehensive plan to address the rising rates of being overweight and obese in Jordanian society. This study highlights the importance of the choice of reference used to measure the prevalence of being underweight, overweight, and obese in Jordan and beyond. It highlights the critical need for accurate early identification, prevention, and treatment of childhood growth abnormalities to mitigate their future impact. Adopting growth references that align more closely with regional health profiles may improve the effectiveness of national nutrition policies, ultimately helping reduce the prevalence of these conditions among Jordanian children and adolescents.

## Figures and Tables

**Table 1:** Centers for Disease Control and Prevention (CDC) classification of children and adolescents’ nutritional status.

Nutritional Status	Age
	2–20 Years
Underweight	BMI-for-age value below the 5th percentile line on the CDC BMI-for-age growth chart
Overweight	BMI-for-age value equal to or above the 85th percentile but below the 95th percentile line on the CDC BMI-for-age growth chart
Obese	BMI-for-age value at or above the 95th percentile line on the CDC BMI-for-age growth chart

**Table 2:** World Health Organization (WHO) classification of children and adolescents’ nutritional status.

Nutritional Status	Age	
	2–5 Years	5–19 Years
Underweight	Weight-for-age below the 3rd percentile line on the WHO BMI-for-age growth chart	Weight-for-age below the 3rd percentile line on the WHO BMI-for-age growth chart
Overweight	BMI-for-age value above the 97th percentile but below the 99.9th percentile line on the WHO BMI-for-age growth chart	BMI-for-age value above the85th percentile but below the 97th percentile line on the WHO BMI-for-age growth chart
Obese	BMI-for-age value above the 99.9th percentile line on the WHO BMI-for-age growth chart	BMI-for-age value above the 97th percentile line on the WHO BMI-for-age growth chart

**Table 3:** Mean and standard deviation of height, weight, BMI, and age for different age groups across 2017–2023.

Year		2–5 Years Sex		6–11 Years Sex		12–19 Years Sex	
		Male	Female	Male	Female	Male	Female
		Mean (SD)	Mean (SD)	Mean (SD)	Mean (SD)	Mean (SD)	Mean (SD)
2017	Height (cm)	90.7 (5.7)	89.7 (6.0)	122.1 (11.1)	123.2 (12.2)	146.5 (12.6)	150.5 (11.5)
	Weight (Kg)	13.4 (1.9)	13.0 (2.0)	25.4 (8.3)	26.4 (10.0)	41.9 (14.0)	49.8 (15.6)
	BMI (kg/m^2^)	16.3 (1.8)	16.1 (1.9)	16.7 (3.2)	16.9 (3.8)	19.1 (4.0)	21.5 (4.6)
	Age (Yrs)	2.8 (0.8)	2.9 (0.8)	8.1 (1.5)	8.3 (1.5)	13.8 (1.9)	15.0 (2.6)
2019	Height (cm)	89.8 (5.8)	89.2 (6.0)	121.3 (11.5)	123.3 (12.6)	144.8 (12.4)	150.3 (11.9)
	Weight (Kg)	13.3 (1.9)	12.9 (2.0)	25.0 (8.6)	27.0 (10.5)	41.0 (13.8)	50.3 (16.1)
	BMI (kg/m^2^)	16.5 (1.9)	16.2 (2.0)	16.6 (3.4)	17.2 (4.0)	19.1 (4.2)	21.7 (4.7)
	Age (Yrs)	2.6 (0.7)	2.7 (0.8)	8.1 (1.6)	8.4 (1.6)	13.6 (1.8)	15.1 (2.7)
2021	Height (cm)	89.8 (5.8)	89.4 (6.2)	123.3 (11.4)	126.2 (11.9)	144.6 (11.3)	150.4 (11.5)
	Weight (Kg)	13.4 (1.7)	13.0 (1.9)	26.2 (9.3)	28.8 (10.4)	40.1 (12.7)	49.8 (15.8)
	BMI (kg/m^2^)	16.6 (2.0)	16.3 (2.2)	16.8 (3.5)	17.7 (4.0)	18.8 (4.0)	21.5 (4.7)
	Age (Yrs)	2.7 (0.7)	2.7 (0.7)	8.4 (1.5)	8.6 (1.5)	13.4 (1.8)	14.8 (2.7)
2023	Height (cm)	90.9 (7.1)	89.7 (6.6)	122.9 (11.1)	126.9 (12.1)	144.8 (11.5)	150.2 (11.9)
	Weight (Kg)	13.6 (2.3)	13.1 (2.1)	26.00 (9.2)	29.4 (11.0)	40.0 (12.8)	49.7 (15.7)
	BMI (kg/m^2^)	16.5 (1.9)	16.3 (2.1)	16.8 (3.6)	17.8 (4.1)	18.7 (3.9)	21.5 (4.6)
	Age (Yrs)	2.8 (0.8)	2.8 (0.7)	8.3 (1.5)	8.6 (1.5)	13.24 (1.6)	14.4 (2.7)
Total	Height (cm)	90.3 (6.0)	89.5 (6.1)	122.4 (11.2)	124.9 (12.3)	145.3 (12.0)	150.4 (11.7)
	Weight (Kg)	13.4 (1.9)	12.9 (2.0)	25.6 (8.8)	27.9 (10.5)	40.8 (13.4)	49.9 (15.7)
	BMI (kg/m^2^)	16.4 (1.9)	16.2 (2.0)	16.7 (3.4)	17.4 (4.0)	18.9 (4.0)	21.6 (4.7)
	Age (Yrs)	2.7 (0.7)	2.8 (0.8)	8.2 (1.5)	8.45 (1.5)	13.5 (1.8)	14.8 (2.7)

**Table 4:** Prevalence of being underweight, normal weight, overweight, and obese, according to CDC and WHO standards, 2017–2023.

Years			2–5		6–11		12–19	
			Sex		Sex		Sex	
			Male	Female	Male	Female	Male	Female
			Count (N%)	Count (N%)	Count (N%)	Count (N%)	Count (N%)	Count (N%)
2017	CDC	Underweight	239 (11.7%)	236 (12.4%)	506 (14.8%)	614 (15.8%)	702 (20.8%)	434 (8.4%)
		Normal	1340 (65.8%)	1214 (63.9%)	2270 (66.2%)	2462 (63.3%)	2106 (62.5%)	3394 (65.9%)
		Overweight	248 (12.2%)	243 (12.8%)	303 (8.8%)	359 (9.2%)	322 (9.6%)	912 (17.7%)
		Obese	210 (10.3%)	206 (10.8%)	348 (10.2%)	457 (11.7%)	241 (7.1%)	412 (8%)
	WHO	Underweight	180 (8.8%)	173 (9.1%)	469 (13.7%)	553 (14.2%)	435 (12.9%)	311 (6%)
		Normal	1622 (79.6%)	1504 (79.2%)	2150 (62.7%)	2328 (59.8%)	2248 (66.7%)	3278 (63.6%)
		Overweight	186 (9.1%)	192 (10.1%)	383 (11.2%)	499 (%)	412 (%)	1029 (%)
		Obese	49 (2.4%)	30 (1.6%)	425 (12.4%)	512 (13.2%)	276 (8.2%)	534 (10.4%)
2019	CDC	Underweight	235 (13.1%)	261 (12.8%)	265 (14.6%)	295 (13.9%)	380 (19.5%)	223 (7.4%)
		Normal	1126 (62.8%)	1353 (66.6%)	1171 (64.7%)	1319 (62%)	1201 (61.6%)	1919 (63.9%)
		Overweight	232 (12.9%)	211 (10.4%)	169 (9.3%)	221 (10.4%)	170 (8.7%)	588 (19.6%)
		Obese	200 (11.2%)	208 (10.2%)	205 (11.3%)	293 (13.8%)	199 (10.2%)	273 (9.1%)
	WHO	Underweight	123 (6.9%)	149 (7.3%)	270 (14.9%)	309 (14.5%)	264 (13.5%)	160 (5.3%)
		Normal	1412 (78.8%)	1648 (81.1%)	1113 (61.5%)	1223 (57.5%)	1274 (65.3%)	1848 (61.5%)
		Overweight	201 (11.2%)	182 (9%)	197 (10.9%)	293 (13.8%)	200 (10.3%)	648 (21.6%)
		Obese	57 (3.2%)	54 (2.7%)	230 (12.7%)	303 (14.2%)	212 (10.9%)	347 (11.6%)
2021	CDC	Underweight	77 (13.3%)	81 (14.4%)	289 (14.5%)	325 (11.2%)	494 (17.7%)	338 (8.1%)
		Normal	360 (62.2%)	345 (61.5%)	1265 (63.6%)	1689 (58.4%)	1785 (63.8%)	2611 (62.5%)
		Overweight	67 (11.6%)	63 (11.2%)	187 (9.4%)	371 (12.8%)	262 (9.4%)	798 (19.1%)
		Obese	75 (13%)	72 (12.8%)	247 (12.4%)	508 (17.6%)	256 (9.2%)	430 (10.3%)
	WHO	Underweight	35 (6%)	39 (7%)	340 (17.1%)	305 (10.5%)	355 (12.7%)	266 (6.4%)
		Normal	447 (77.2%)	447 (79.7%)	1160 (58.4%)	1637 (56.6%)	1901 (68%)	2570 (61.5%)
		Overweight	73 (12.6%)	52 (9.3%)	227 (11.4%)	432 (14.9%)	289 (10.3%)	847 (20.3%)
		Obese	24 (4.1%)	23 (4.1%)	261 (13.1%)	519 (17.9%)	252 (9%)	494 (11.8%)
2023	CDC	Underweight	74 (12.2%)	74 (12.9%)	294 (13.5%)	275 (8.2%)	382 (15.9%)	229 (5.9%)
		Normal	392 (64.6%)	359 (62.8%)	1398 (64.2%)	2041 (61%)	1503 (62.7%)	2351 (60.5%)
		Overweight	69 (11.4%)	61 (10.7%)	180 (8.3%)	409 (12.2%)	256 (10.7%)	846 (21.8%)
		Obese	72 (11.9%)	78 (13.6%)	304 (14%)	622 (18.6%)	255 (10.6%)	460 (11.8%)
	WHO	Underweight	32 (5.3%)	39 (6.8%)	359 (16.5%)	323 (9.7%)	306 (12.8%)	180 (4.6%)
		Normal	476 (78.4%)	446 (78%)	1277 (58.7%)	1935 (57.8%)	1630 (68%)	2334 (60.1%)
		Overweight	81 (13.3%)	68 (11.9%)	233 (10.7%)	475 (14.2%)	247 (10.3%)	903 (23.2%)
		Obese	18 (3%)	19 (3.3%)	307 (14.1%)	614 (18.3%)	213 (8.9%)	469 (12.1%)
Total	CDC	Underweight	625 (12.5%)	652 (12.9%)	1354 (14.4%)	1509 (12.3%)	1958 (18.6%)	1224 (7.5%)
		Normal	3218 (64.2%)	3271 (64.6%)	6104 (64.9%)	7511 (61.3%)	6595 (62.7%)	10,275 (63.4%)
		Overweight	616 (12.3%)	578 (11.4%)	839 (8.9%)	1360 (11.1%)	1010 (9.6%)	3144 (19.4%)
		Obese	557 (11.1%)	564 (11.1%)	1104 (11.7%)	1880 (15.3%)	951 (9%)	1575 (9.7%)
	WHO	Underweight	370 (7.4%)	400 (7.9%)	1438 (15.3%)	1490 (12.2%)	1360 (12.9%)	917 (5.7%)
		Normal	3957 (78.9%)	4045 (79.9%)	5700 (60.6%)	7123 (58.1%)	7053 (67.1%)	10,030 (61.8%)
		Overweight	541 (10.8%)	494 (9.8%)	1040 (11.1%)	1699 (13.9%)	1148 (10.9%)	3427 (21.1%)
		Obese	148 (3%)	126 (2.5%)	1223 (13%)	1948 (15.9%)	953 (9.1%)	1844 (11.4%)

**Table 5:** Regression of the variables associated with being overweight and obese according to WHO standards.

Variables	2–5 Years				6–11 Years				12–19 Years				Total				
	(OR)	*p*	95% C.I. for OR		(OR)	*p*	95% C.I. for OR		(OR)	*p*	95% C.I. for OR		Variables	(OR)	*p*	95% C.I. for OR	
			Lower	Upper			Lower	Upper			Lower	Upper				Lower	Upper
Year of anthropometric measures	1.13	0.03	1.01	1.27	0.77	<0.01	0.73	0.82	0.60	<0.01	0.57	0.64	Year of anthropometric measures	1.04	<0.01	1.03	1.05
Male vs. female	0.78	<0.01	0.72	0.85	1.10	<0.01	1.08	1.12	1.12	<0.01	1.10	1.13	Male vs. female	0.66	<0.01	0.64	0.69
Child/adolescent age (Yrs)	1.06	<0.01	1.03	1.09	1.04	<0.01	1.02	1.05	1.03	<0.01	1.02	1.04	Age group	1.35	<0.01	1.32	1.39

OR: odds ratio, *p*: *p*-value.

**Table 6:** Regression of the variables associated with being overweight and obese according to CDC standards.

Variables	2–5 Years				6–11 Years				12–19 Years				Total	Total			
	(OR)	*p*	95% C.I. for OR		(OR)	*p*	95% C.I. for OR		(OR)	*p*	95% C.I. for OR		Variables	(OR)	*p*	95% C.I. for OR	
			Lower	Upper			Lower	Upper			Lower	Upper				Lower	Upper
Year of anthropometric measures	1.05	0.33	0.96	1.15	0.75	<0.01	0.70	0.80	0.63	<0.01	0.60	0.67	Year of anthropometric measures	1.06	<0.01	1.05	1.06
Male vs. female	0.97	0.35	0.91	1.03	1.08	<0.01	1.05	1.098	1.10	<0.01	1.08	1.11	Male vs. female	0.69	<0.01	0.67	0.72
Child/adolescent age (years)	1.01	0.49	0.99	1.03	1.07	<0.01	1.05	1.08	1.065	<0.01	1.05	1.08	Age group	1.02	0.167	0.99	1.05

OR: odds ratio, *p*: *p*-value.

## Data Availability

The dataset supporting the conclusions of this article is available in the Zenodo repository: https://doi.org/10.5281/zenodo.14442564.
